# Potential of Chitinolytic *Serratia marcescens* Strain JPP1 for Biological Control of *Aspergillus parasiticus* and Aflatoxin

**DOI:** 10.1155/2013/397142

**Published:** 2013-06-20

**Authors:** Kai Wang, Pei-sheng Yan, Li-xin Cao, Qing-long Ding, Chi Shao, Teng-fei Zhao

**Affiliations:** School of Marine Science and Technology, Harbin Institute of Technology, Weihai 264209, China

## Abstract

*Serratia marcescens* strain JPP1 was isolated from peanut hulls in Huai'an city, Jiangsu Province, China. Its potential to inhibit the mycelial growth of *Aspergillus parasiticus* and the subsequent aflatoxin production was evaluated. The strain JPP1 could produce chitinase to degrade fungal cell walls, which was the main mechanism of strain JPP1 for biocontrol. Scanning electron microscopy of fungi treated with the crude chitinase revealed abnormal morphological changes. While the strain was grown in the peanut hulls-based medium, the chitinase activity reached 7.39 units. RT-PCR analysis showed that the crude chitinase repressed the transcription of genes involved in the aflatoxin gene cluster, such as aflR, aflC (*pks*L1), and aflO (*dmt*A) genes. By visual agar plate assay and tip culture method, the strain JPP1 exhibited remarkable inhibitory effect on mycelia growth (antifungal ratio >95%) and subsequent aflatoxin production (antiaflatoxigenic ratio >98%). An *in vitro* assay with seed coating agent of bacterial suspension showed that strain JPP1 effectively reduced fungal growth and subsequent aflatoxin production on peanut seeds, and its antagonistic effect was superior to the common agricultural fungicide of carbendazim. These characteristics suggest that *S. marcescens* JPP1 strain could potentially be utilized for the biological control of phytopathogenic fungi and aflatoxin in Chinese peanut main producing areas.

## 1. Introduction 

Aflatoxins (AFs) are highly toxic and carcinogenic secondary metabolites produced by three species of* Aspergillus *[1]*. Aspergillus flavus* and *Aspergillus parasiticus* are the major contaminants of agricultural commodities [2–4].* Aspergillus flavus* isolates produce only B-aflatoxins, while *A. parasiticus* produce both B- and G-aflatoxins. Aflatoxin B_1_ is the most commonly occurring and known to be teratogenic and carcinogenic for humans and animals. Contamination of peanuts with AFs is a worldwide problem that affects food safety and agricultural economies. In fact, the incidence of liver cancer is much higher in regions with high endemic AFs contamination, such as the developing countries in Southeast Asia and southern Africa. Most developed countries have adopted regulations limiting the concentration of AFs in food and feed to 20 *μ*g/Kg or less [5].

In order to eliminate AFs contamination, biological control is one of the most promising techniques for controlling preharvest crop infection by aflatoxin-producing fungi [6]. Therefore, many microorganisms have been tested for antifungal activity [7, 8] and for aflatoxin inhibitory activity [9–12]. Endophytic bacteria live inside the plant tissues and do not cause morphological changes or visible damage, even benefiting the host by growth-promoting effect. Therefore, they have attracted more attention as novel resources for plant diseases [13].

Chitin is a major constituent of the cell walls of many fungi, insect exoskeletons, and crustacean shells. Chitinolytic bacteria as biocontrol agents have showed potential antagonistic activity against pathogenic fungi by degrading the cell walls [14]. *Serratia marcescens *has been reported producing multiple chitinases (ChiA, ChiB, and ChiC) [15], and these enzymes are able to degrade the chitin in the cell walls of fungi and the exoskeletons of insects. *Serratia marcescens *strain B2 has been reported as biocontrol agent of *Rhizoctonia solani*, *Fusarium oxysporum,* and *Botrytis cinerea* and rice sheath blight [16, 17]. However, there is no report performing on endophytic bacteria isolated from peanut hulls against the growth of *A. parasiticus* and aflatoxin production to our knowledge.

In this study, an endophytic bacterium isolated from peanut hulls was identified and investigated on its potential biocontrol activity against pathogenic fungi and AFs. The visual agar plate assay and tip culture method were used to determine the range and extent of antifungal phenotypes. A peanut hulls-based medium was chosen for chitinase production and exhibited remarkable antagonistic activity for AFs. Scanning electron microscopy of fungi treated with the cell-free culture filtrate (CCF) of strain JPP1 revealed morphological changes. The relationship between the antagonistic ability against AFs and chitinase activity was determined. RT-PCR was performed to demonstrate the effect of chitinase produced by strain JPP1 on the expression of some aflatoxin biosynthetic genes. A biological seed coating agent was selected and its antagonistic ability against AFs on peanut seeds was also studied. The results obtained will aid in using the strain JPP1 for formulating a biofungicide effective in diminishing the use of chemical fungicides in the control of phytopathogenic fungi and aflatoxin.

## 2. Materials and Methods

### 2.1. Isolation of Biocontrol Bacterium

Sampling site was located in Huai'an city, Jiangsu Province, China. The sample was collected from peanut plant and kept in a cooler. The biocontrol bacterium was isolated from the peanut hulls. Peanut pods were washed to release the attached soil with sterile water, then surface-sterilized with 3.0% (w/v) NaOCl for 10 min, and extensively washed with sterile water. The peanut hulls were separately partitioned into 6 pieces and placed on potato dextrose agar (PDA) medium. The plates were incubated at 28°C for 4 days.

### 2.2. Media Composition

PDA medium (% w/v) is as follows: potatoes, 20; dextrose, 2; agar, 2. GY medium (% w/v): glucose, 2; yeast extract, 0.5; agar, 2; pH in nature. PGY medium is as follows: peanut hulls were dried at 40°C and then ground. The ground peanut hulls were boiled with water for 1 h at the final concentration of 2.5% and then centrifuged at 6,600 ×g at room temperature for 5 min. The supernatant was supplemented with 2% glucose and 0.5% yeast extract, then autoclaved for 20 min at 121°C, pH in nature. Chitinase medium: PGY medium was supplemented with 1% colloidal chitin.

### 2.3. Biocontrol Bacterium Screening

The visual agar plate assay was used to select the biocontrol bacterium with the mutant *A. parasiticus* NFRI-95, which was obtained by UV irradiation of aflatoxigenic *A. parasiticus* SYS-4. The mutant NFRI-95 strain could accumulate norsolorinic acid (NA), an orange-red pigment precursor of aflatoxin. The spores were inoculated in a line at the center of solid GY medium. Then an aliquot of the bacterium culture was inoculated in a line 1.5 cm from the centerline. After 4 days of incubation at 28°C, the antagonistic effects of the bacterium on either NA accumulation in the mycelium or the fungal growth were observed. One of the selected bacteria which showed remarkable antagonistic activity was designated JPP1.

### 2.4. Identification of the Biocontrol Bacterium

The morphological characteristics of the selected JPP1 bacterium, which had been cultured on PDA media at 28°C, were observed by scanning electron microscopy (SEM). Gram staining, casein hydrolysis, catalase reactivity, and citrate degradation were also determined. Final identification of the bacterium was performed by 16S rRNA sequence analysis. Genomic DNA from strain JPP1 was extracted using a bacterial genomic DNA FastPrep Extraction Kit (Sangon Biotech, China). Polymerase chain reaction (PCR) amplification of the nearly full-length 16S rRNA gene was performed using the universal primers 16F27 and 16R1522. PCR was performed under the following conditions: 95°C for 5 min, followed by 94°C for 1 min, 55°C for 1 min, and 72°C for 2 min for 35 cycles and by a final 10 min extention at 72°C. PCR sequencing was performed at Sangon Biotech, Shanghai. Similarity searches of all the sequences were performed in the GenBank database using BLASTn [18]. The sequences alignments were performed using CLUSTAL_X [19], and a phylogenetic tree was constructed from evolutionary distances using the neighbor-joining method of MEGA 5 program package [20]. The 16S rRNA gene sequence of the strain JPP1 was submitted to NCBI GenBank Database under the accession number JQ308601.

### 2.5. Quantitative Antagonistic Test against AFs

The quantitative antagonism of the JPP1 bacterium against fungal growth and AFs production was determined by tip culture method [21]. The selected JPP1 bacterium was inoculated in 3 mL PGY medium. After being shaken on a rotary shaker (30°C, 140 rpm) for 12 h, the JPP1 culture was transferred into 300 mL chitinase medium to cultivate at 30°C for 6 days. After being centrifuged at 4,224 ×g for 10 min at 4°C, the supernatant was filtered through a 0.22 *μ*m pore size filter to yield the CCF. Then 700 *μ*L CCF was added to a 5 mL tip as culture vessel, and the control tip was used without CCF. Each treatment tip was inoculated separately with 10 *μ*L of *A. parasiticus* NFRI-95 spores. After incubation at 26°C for 6 days, the mycelia and culture medium were separated by centrifugation at 96 ×g. The mycelial was weighed by an electronic balance with accuracy of 0.1 mg and calculated for antifungal ratio relative to control. To detect precursor of the AFs, the NA accumulation was determined by extracting the red-orange pigment of the mycelia in the tip. The extraction was at room temperature for 2 h with a solution containing 1 mol/L NaOH and methanol (9 : 1, v/v). The extract was centrifuged at 4,000 ×g for 5 min and the absorbance was taken at 560 nm. Thus, the extent of bacterial inhibition on NA accumulation was determined relative to control. Data is expressed as mean ± SD of three or more experiments.

### 2.6. SEM of Fungal Morphological Changes

SEM was carried out to reveal the morphological changes of the fungi treated with the tip culture method. Fungal samples were fixed by immersion in 3% (v/v) glutaraldehyde for 4 h, washed three times in 0.1 mol/L phosphate buffer pH 7.2. Subsequently they were dehydrated in alcohol at a series of concentrations (30, 50, 70, 80, and 100% v/v) for 10 min resting time each, among which the samples were dehydrated twice in alcohol at concentration of 100% (v/v). Then they were displaced by 50% (v/v) isoamyl acetate once for 10 min and 100% (v/v) isoamyl acetate for 20 min. The materials were dried in a CO_2_ apparatus (Eiko DX-1), sputter-coated with gold (Eiko IB-3), and viewed using a scanning electron microscope (JEOL JSM-840).

### 2.7. Chitinase Activity and Antagonistic Activity

For determining the relationship between the antagonistic ability against AFs and chitinase activity, the JPP1 bacterium was inoculated in chitinase medium at 28°C. After cultivation for 0, 24, 46, 62, 76, 92, 110, and 128 h, the medium was centrifuged at 4,224 ×g for 10 min, and the supernatant was filtered to yield the CCF of crude chitinase. The CCF of JPP1 bacterium was used to determine the antagonistic ability against AFs by tip culture method. Meanwhile the CCF was tested for chitinase activity according to the method of Monreal and Reese detecting N-acetylglucosamine (NAG) as the final product [22]. One unit of chitinase activity was defined as the amount of enzyme that catalysed the release of  1 *μ*mol of NAG per hour at 50°C. Data is expressed as mean ± SD of three experiments.

### 2.8. RT-PCR of the Aflatoxin Biosynthetic Genes

Toxigenic *A. parasiticus *strain of SYS-4 was cultured in the CCF of strain JPP1 supplemented with 2% glucose and 0.5% yeast extract. The control was cultured in GY medium without the chitinase CCF. After the mycelia were cultured for 6 d, total RNA of the mycelia were prepared using the UNIQ-10 Trizol RNA kit (Sangon Biotech, China) and the synthesis of cDNA was performed using the M-MuLV cDNA synthesis kit (Sangon Biotech, China). The primers were aflR-F (5′-CGCGGATCCATGGTTGACCATATCTCCCC) and aflR-R (5′-CCCCAAGCTTCATTCTCGATGCAGGTAATC) for aflR gene (accession no. AF441437), aflC-F (5′-CCAGGACAGCCCTATTCTAG) and aflC-R (5′-GGAGTCCAGTGGTATTCAGC) for the polyketide synthetase gene (accession no. L42766), and aflO-F (5′-ACAAATACCCCTGGCTCAGG) and aflO-R (5′-ACCTGTTCCATCAAATCGTC) for the *O*-methyltransferase I gene (accession no. AB022906). PCR was performed using the cDNA as template under the following conditions: 95°C for 5 min, followed by 95°C for 0.5 min, 54°C for 1 min, and 72°C for 2 min for 40 cycles and by a final 10 min extention at 72°C. TLC was used to verify the aflatoxin production, the solvent system containing chloroform, ethyl acetate, and 90% formic acid (6 : 3 : 1).

### 2.9. The Antagonistic Activity of Biological Seed Coating Agent on Peanuts *In Vitro*

The JPP1 bacterium was used as biological agent and incubated in chitinase medium at 28°C on a rotary shaker (140 rpm) for 48 h. Adjust the concentration of filmogen to yield the peanut biological seed coating agent, with higher film-forming quality. Twelve grams of peanut seeds were distributed in each of 100 mL flasks and autoclaved. Then 3 mL biological seed coating agent was added to each flask and the control was used as medium only. Meanwhile, the common agricultural fungicide of carbendazim was tested for the antagonistic effect. After being diluted 100-fold the carbendazim was added to the peanut seeds according to the amount of 0.4% (v/m). Then all the flasks were kept on a rotary shaker (140 rpm) for 15 min and then dried on sterile condition. All the flasks were then inoculated with 500 *μ*L of *A. parasiticus* NFRI-95 spores (4 × 10^5^ CFU/mL) and incubated at 30°C for 3 days. The growth of *A. parasiticus* was evaluated macroscopically. To determine the quantitative inhibitory effect of biological seed coating agent on precursor of the AFs, the red-orange pigment of the mycelia on peanuts was extracted in the flasks. The 10 mL solution containing 1 mol/L NaOH and methanol (9 : 1, v/v) was added to each flask. The flasks were kept on a rotary shaker (140 rpm) for 30 min and the extract was collected. After three times of extraction, the 30 mL extract was centrifuged at 8,000 ×g for 10 min and the absorbance was taken at 560 nm. Data is expressed as mean ± SD of three experiments.

## 3. Results 

**Figure 1:**
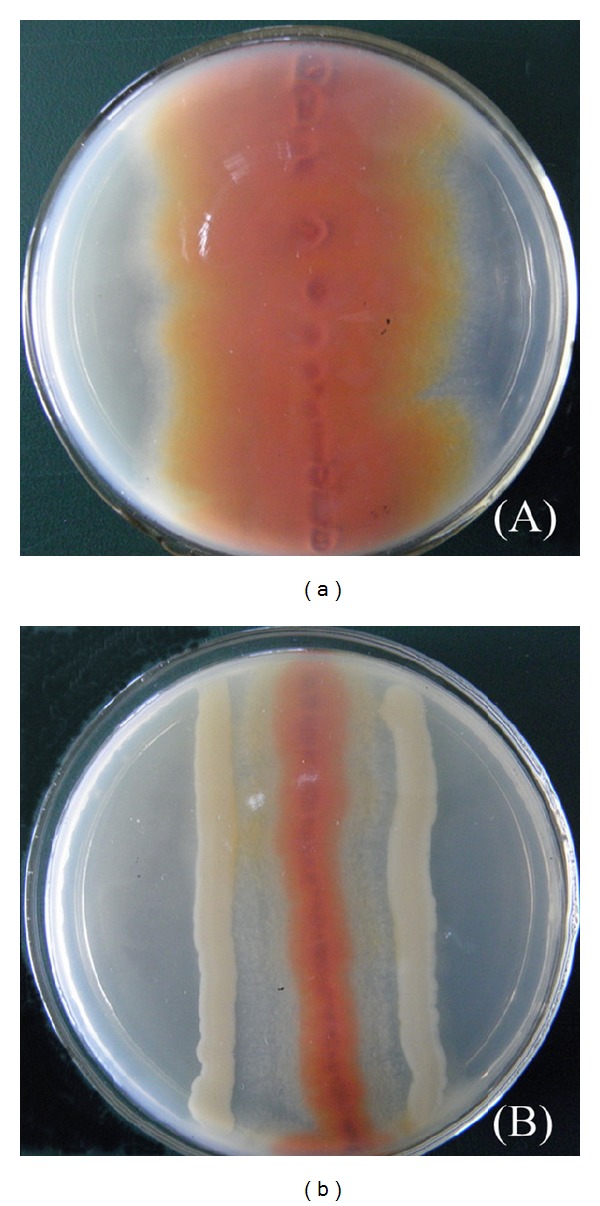
Results of a visual agar plate assay with *Aspergillus parasiticus* NFRI-95 without (a) or with (b) bacterial strain JPP1.

Our laboratory is in the process of building a collection of strains which can potentially be biocontrol agents against AFs. Strain JPP1 was isolated from a peanut plant in Jiangsu Province which is one of the main peanut producing areas in China. By visual agar plate assay, the JPP1 bacterium exhibited inhibitory activity against mycelia growth and AFs production. The inhibitory effect could be observed by the loss of NA accumulation in the fungal mycelium (Figure 1(b)) relative to control (Figure 1(a)).

**Figure 2:**
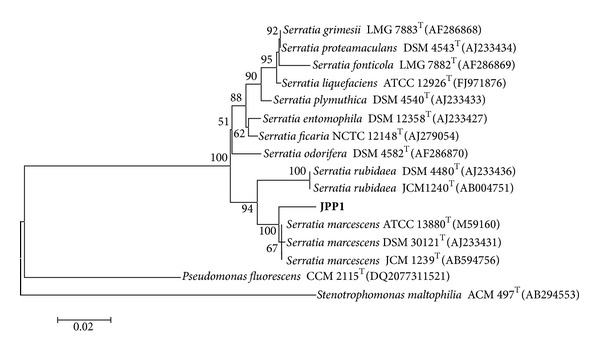
Neighbor-joining tree based on the 16S rRNA sequences of strain JPP1 and some other related representative strains. The numbers on the tree indicate the percentage of bootstrap based on 1,000 replications and are shown for branches with more than 50% support. The scale bar represents 0.02 nucleotide substitution per sequence position.

The JPP1 bacterium was a rod-shaped organism that was gram negative, casein hydrolysis, catalase reactivity, and citrate degradation positive. We determined a 1,489-nucleotide sequence for the 16S rRNA of JPP1 bacterium. The phylogenetic relationship of the strain with those of representative strains of the genus *Serratia* was shown in Figure 2. The phylogenetic tree showed that the JPP1 isolate was clustered within the same group with the type strains ATCC 13880 (M59160), JCM 1239 (AB594756), and DSM 30121 (AJ233431) of *Serratia marcescens* and showed more than 98% sequence similarity values. Therefore, the strain JPP1 was identified as *S. marcescens* and we designated this organism *S. marcescens* JPP1.

**Figure 3:**
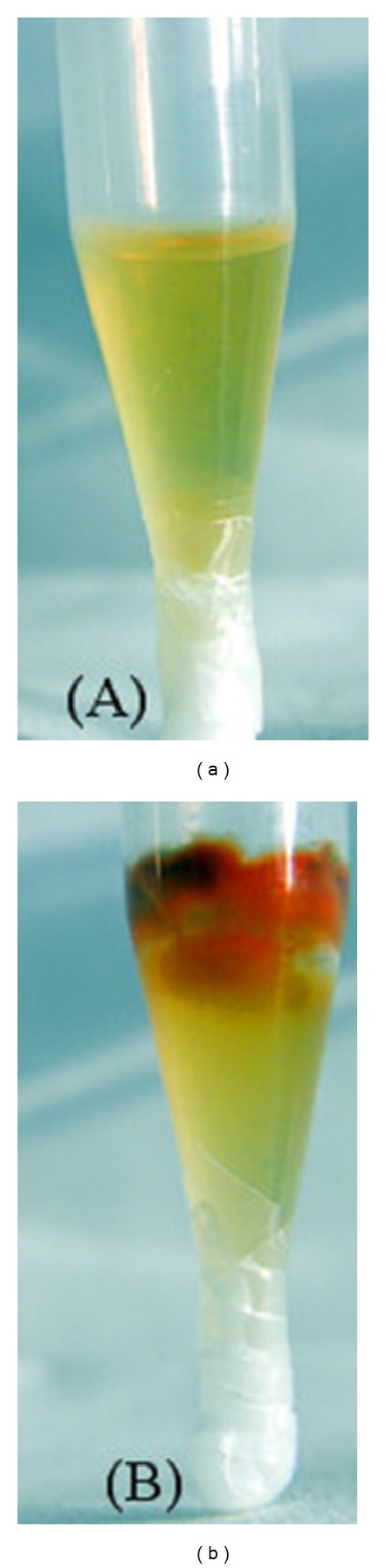
Inhibitory effect of *S. marcescens* JPP1 cell-free culture filtrate on fungal growth and AFs production by tip culture method (a) treated with the cell-free culture filtrate of strain JPP1 (b) control.

By tip culture method the strain JPP1 was tested for quantitative antagonism against mycelia growth and AFs production. The inhibitory effect against mycelia growth and AFs production was directly observed (Figure 3(a)) relative to control (Figure 3(b)). The strain JPP1 exhibited remarkable inhibitory effect on mycelia growth (antifungal ratio >95%) and subsequent aflatoxin production (antiaflatoxigenic ratio >98%) (Table 1).

**Figure 4:**
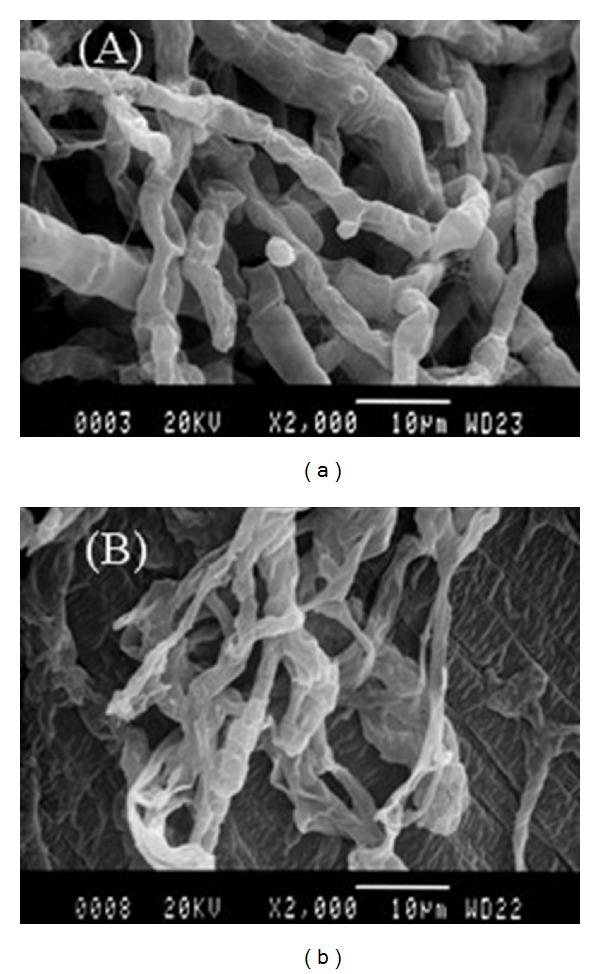
Scanning electron micrographs of mycelia of *Aspergillus parasiticus* NFRI-95 (a) control (b) treated with the cell-free culture filtrate of strain JPP1 bacterium. Scales: 10 *μ*m.

SEM was carried out to reveal the morphological changes of the fungi treated with the CCF of JPP1. SEM analysis showed major damage of the mycelia and the spores of* A. parasiticus* NFRI-95 in the presence of* S. marcescens* JPP1 CCF (Figure 4(b)) relative to control (Figure 4(a)). The mycelia obviously showed abnormal forms suggesting degradation of the fungal cell wall. 

**Figure 5:**
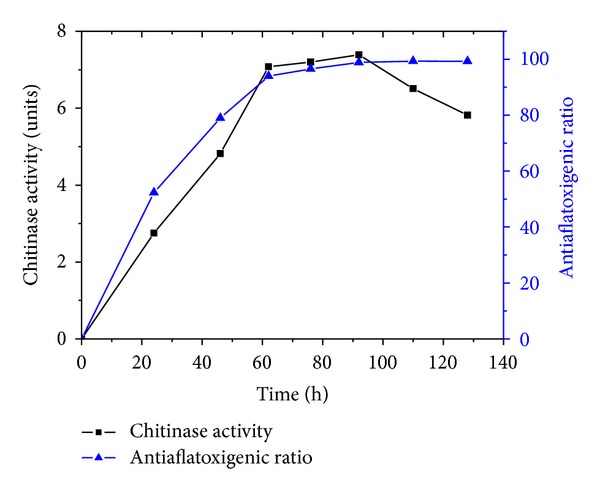
Antagonistic ability against aflatoxins and chitinase activity of *S. marcescens* JPP1.

Since chitinolytic bacteria have showed potential antagonistic activity against pathogenic fungi by degrading the cell walls, the chitinase activity of strain JPP1 was investigated. The relationship between the antagonistic ability against AFs and chitinase activity was also investigated. As shown in Figure 5, the antagonistic ability increased rapidly before 62 h. After 92 h the antiaflatoxigenic ratio reached 98.87% and increased smoothly. Meanwhile, the production of chitinase by strain JPP1 increased and reached the peak level after 92 h. Chitinase activity was not entirely related to the antagonistic ability and growth of strain JPP1 (data not shown). 

**Figure 6:**
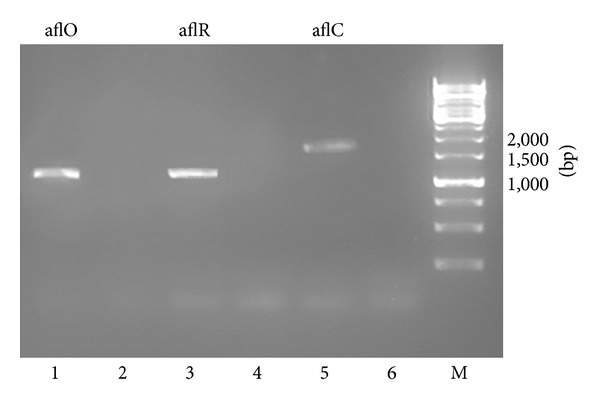
RT-PCR of the aflatoxin-related genes. *A. parasiticus *strain of SYS-4 was cultured and treated with the chitinase CCF of strain JPP1 (lanes 2, 4, and 6); the control was in the absence of CCF (lanes 1, 3, and 5).

**Figure 7:**
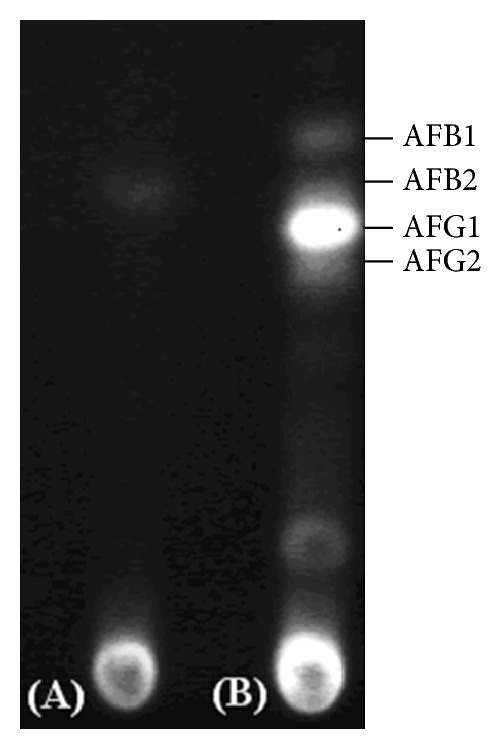
Inhibitory effect of strain JPP1 CCF on aflatoxins production by TLC (a) treated with the cell-free culture filtrate of strain JPP1 (b) control.

RT-PCR was performed to demonstrate the effect of chitinase produced by strain JPP1 on the expression of the aflatoxin biosynthetic genes, aflR, aflC (*pks*L1), and aflO (*dmt*A). Expression of the aflatoxin-related genes aflR, aflC, and aflO was detected in the absence of the chitinase CCF. However, expression of these genes was completely inhibited by the CCF of strain JPP1 (Figure 6). Inhibition of aflatoxin production by the CCF was also confirmed by TLC, which is the common method for aflatoxin detection. TLC showed that there was no obvious aflatoxins accumulation (Figure 7(a)) relative to control (Figure 7(b)).

**Figure 8:**
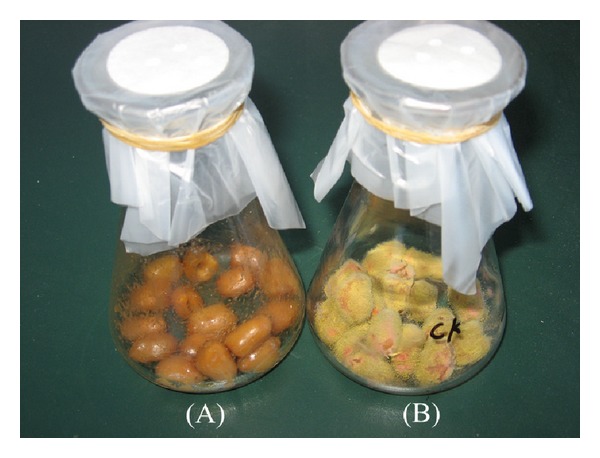
Antifungal activity of the biological seed coating agent with *S. marcescens* JPP1 on peanuts (a) treated with the biological seed coating agent (b) control.

The formulation of seed coating agent was determined; 4% polyvinyl alcohol and 4% carboxymethyl cellulose sodium (4 : 1, v/v) were mixed to yield the filmogen. Then the whole JPP1 bacterium medium (approximately 1 × 10^9^ CFU/mL) and the filmogen (1 : 4, v/v) were mixed to yield the peanut biological seed coating agent.* In vitro* the antagonistic activity of biological seed coating agent on peanuts was also determined. Compared with the blank control (Figure 8(b)), the biological seed coating agent of strain JPP1 significantly reduced mycelial growth (Figure 8(a)), and the antiaflatoxigenic ratio of AFs production was 92.58%, superior to the antiaflatoxigenic ratio (82.40%) of common agricultural fungicide of carbendazim.

## 4. Discussion

To search for biocontrol agent against AFs, the strain JPP1 was isolated with antagonistic activity against* A. parasiticus* NFRI-95. In the present study the strain JPP1 was identified as *S. marcescens *based on biochemical and genetic characteristics. In fact, most of the biocontrol strains of the genus *Serratia* were reported belonging to *S. marcescens *[23]. This species is well known with the potential to control pathogenic fungi by producing hydrolytic enzymes to degrade fungal cell walls, and most of them produce multiple chitinases. *S. marcescens *strain B2 has been reported as biocontrol agent of *Rhizoctonia solani*, *Fusarium oxysporum*, and *Botrytis cinerea* and rice sheath blight. However, few studies have been reported on *S. marcescens* as endophytic bacterium against the growth of *A. parasiticus* and aflatoxin production. In the present study *S. marcescens* JPP1 was isolated from peanut hulls and its CCF significantly inhibited mycelia growth and subsequent aflatoxin production. 

In the present study the main mechanism of strain JPP1 for biocontrol against the growth of *A. parasiticus* and aflatoxin production was also determined. The strain* S. marcescens* JPP1 could produce chitinase to degrade phytopathogenic fungal cell walls. SEM was carried out to reveal the morphological changes, and the mycelia obviously showed abnormal forms suggesting degradation of the fungal cell walls because of the chitinase produced by the strain JPP1. The chitinolytic activity depends on various factors, such as the number, amounts, and activity of the enzymes. In our study, the chitinase activity of 7.39 units was determined while the strain JPP1 was grown in the PGY medium supplemented with little chitin. The relationship between the antagonistic ability against AFs and chitinase activity was also investigated. The results exhibited that chitinase activity was not entirely related to the antagonistic ability against AFs production. Although the chitinase to degrade fungal cell walls was the main mechanisms of strain JPP1 for biocontrol, it is probable that there are other metabolites, such as prodigiosin with chitinases to produce synergistic antagonist effects. RT-PCR results showed that the chitinase CCF repressed the transcription of genes involved in the aflatoxin gene cluster, such as aflR, aflC (*pks*L1), and aflO (*dmt*A) genes. The aflR gene encodes a transcription factor that positively regulates expression of the structural genes [24], while the absence of aflR expression caused the lack of expression of the structural genes aflC and aflO [25].

In the present study, a biological seed coating agent with *S. marcescens* JPP1 was determined and all the materials were environmental friendly. The selected formulation has a good film-forming property and guarantees enough water and oxygen for seeds during normal germination. Moreover, *S. marcescens* JPP1 could survive for more than 14 d mixed with the filmogen (data not shown). *In vitro* the biological seed coating agent of strain JPP1 significantly reduced mycelial growth and AFs production, and its antagonistic effect was superior to the common agricultural fungicide of carbendazim. *S. marcescens* is a ubiquitous organism in water, soil, and food; thus the bacterium may survive well in the field. Certainly further studies of application in situ will be necessary to determine the quantitative inhibitory effect against AFs production on peanuts. Nevertheless, this study indicates that *S. marcescens* JPP1 can potentially be developed as new biocontrol agent for formulating a biofungicide effective in diminishing the use of chemical fungicides in the control of phytopathogenic fungi and aflatoxin in peanut.

## Figures and Tables

**Table 1:** Inhibitory effect of strain JPP1 on mycelia growth and AFs production.

Test strain	Average fresh weight of mycelium (g)	Antifungal ratio (%)	Average of OD560	Antiaflatoxigenic ratio (%)
JPP1	0.0029 ± 0.0003**	95.03	0.020 ± 0.002**	98.94
CK	0.0584 ± 0.0006		1.895 ± 0.005	

Inhibitory effect of strain JPP1 on mycelia growth and AFs production was determined by the tip culture method [20].

**P < 0.01 relative to control.
